# Supplementary figures and images for: miRNA-21 regulates CD69 and IL-10 expression in canine leishmaniasis
Source: PLoS One. 2022 Mar 24;17(3):e0265192. doi: 10.1371/journal.pone.0265192 (PMC8947396; doi:10.1371/journal.pone.0265192)

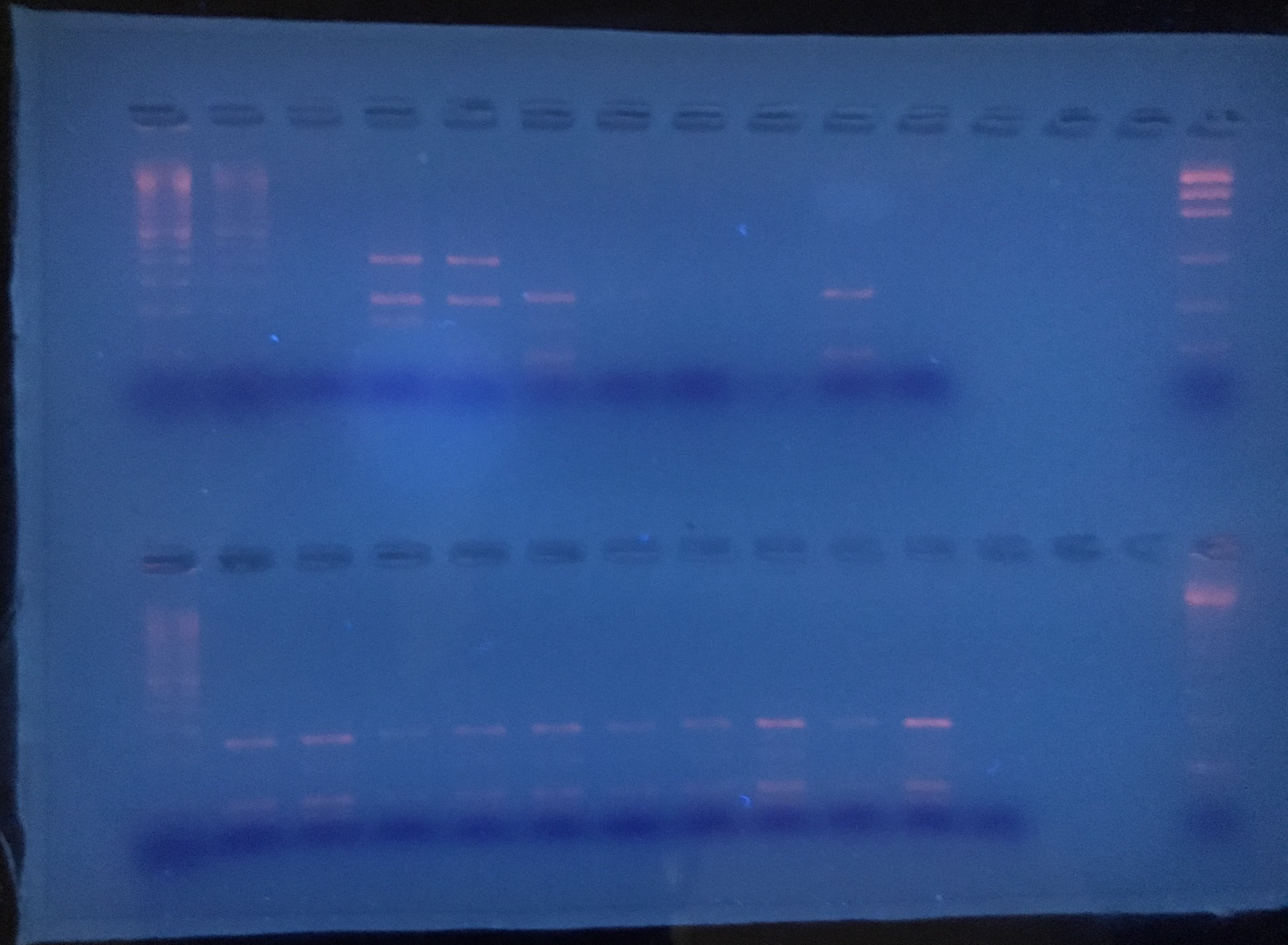

Supplement: S1 Fig — Restriction fragment length polymorphism analysis of ITS1-PCR fragments amplified from DNA samples using Hae III enzyme. NC: Negative control (water); M: molecular marker (123 bp); La: Leishmania amazonensis (IOC / L0575-MHOM / BR / 1967 / PH8); Lb: Leishmania braziliensis (IOC / L0566-MHOM / BR / 1975 / M2903); Li: Leishmania infantum (IOC / L0575-MHOM / BR / 2002 / LPC-RPV); C1 to C5: control group; CanL 1 to CanL10: CanL group. CanL sample profiles were identical to L. infantum. (TIFF) [file pone.0265192.s001.tiff]

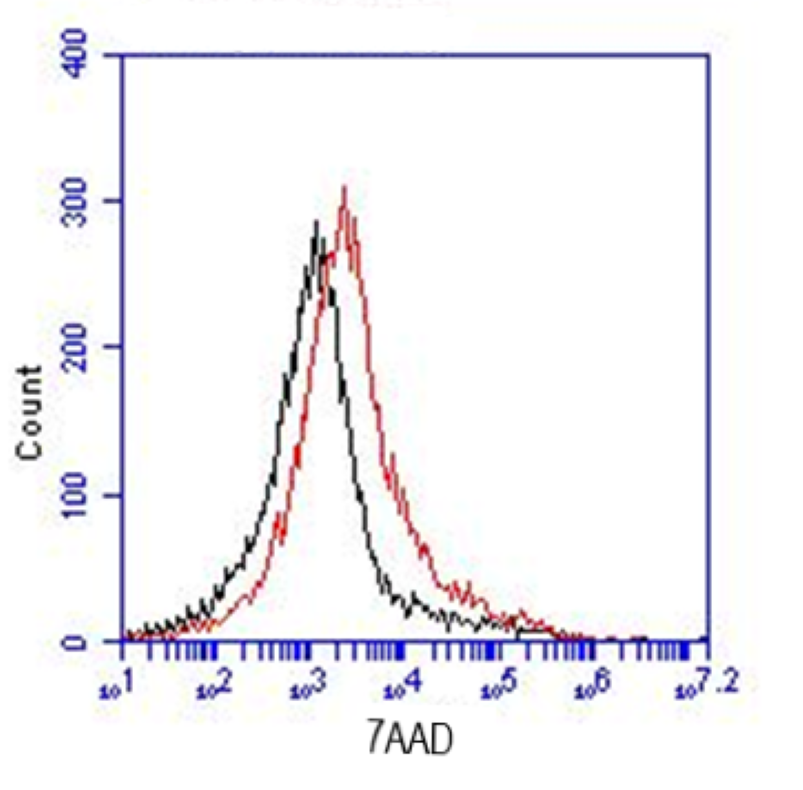

Supplement: S2 Fig — Representative histogram obtained from flow cytometry analysis. The red line represents the cells cultured with reagent Cell Death, and the black line represents the cells cultures without any transfection reagent. Cells were cultured for 48 h at 37°C and 5% CO2. (TIFF) [file pone.0265192.s002.tiff]

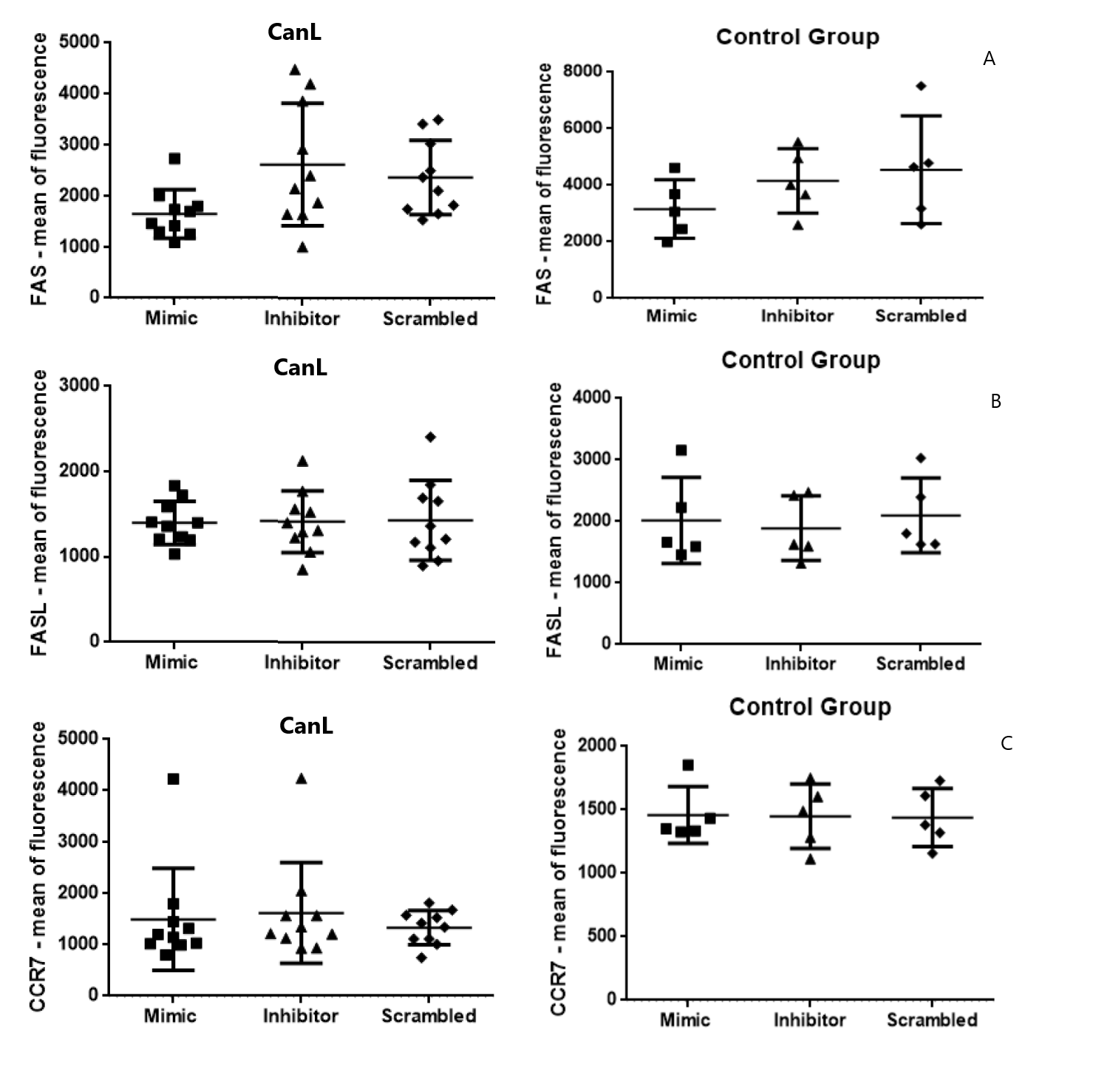

Supplement: S3 Fig — Expression of FAS (A), FASL (B) and CCR7 (C) proteins in splenic leukocytes of the CanL and Control groups. Splenic leukocytes of dogs naturally infected by L. infantum and healthy dogs were transfected with scrambled, miR-21 mimic, and miR-21 inhibitor, all in the presence of Hiperfect, following 48 h in culture at 37°C and 5% CO2. Data are presented as median ± min-max. The asterisk indicates significant differences (Friedman’s multiple comparison test, * p < 0.05). (TIFF) [file pone.0265192.s003.tiff]

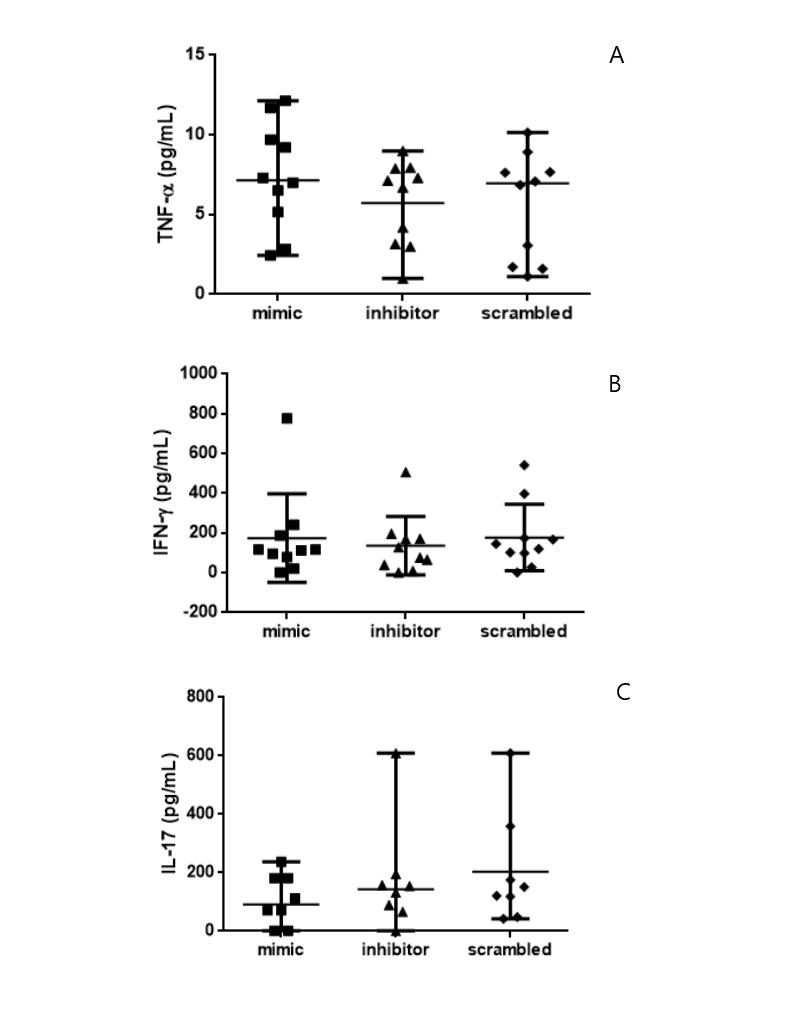

Supplement: S4 Fig — Splenic leukocytes from the CanL group were transfected with miR-21 mimics and inhibitors, and after 48 hours, cytokine concentrations were measured by capture ELISA in cell culture supernatants. Data represent the median values + min-max. Asterisks represent significance (p < 0.05) by the Friedman Test with the Dunn multiple comparisons. (TIFF) [file pone.0265192.s004.tiff]

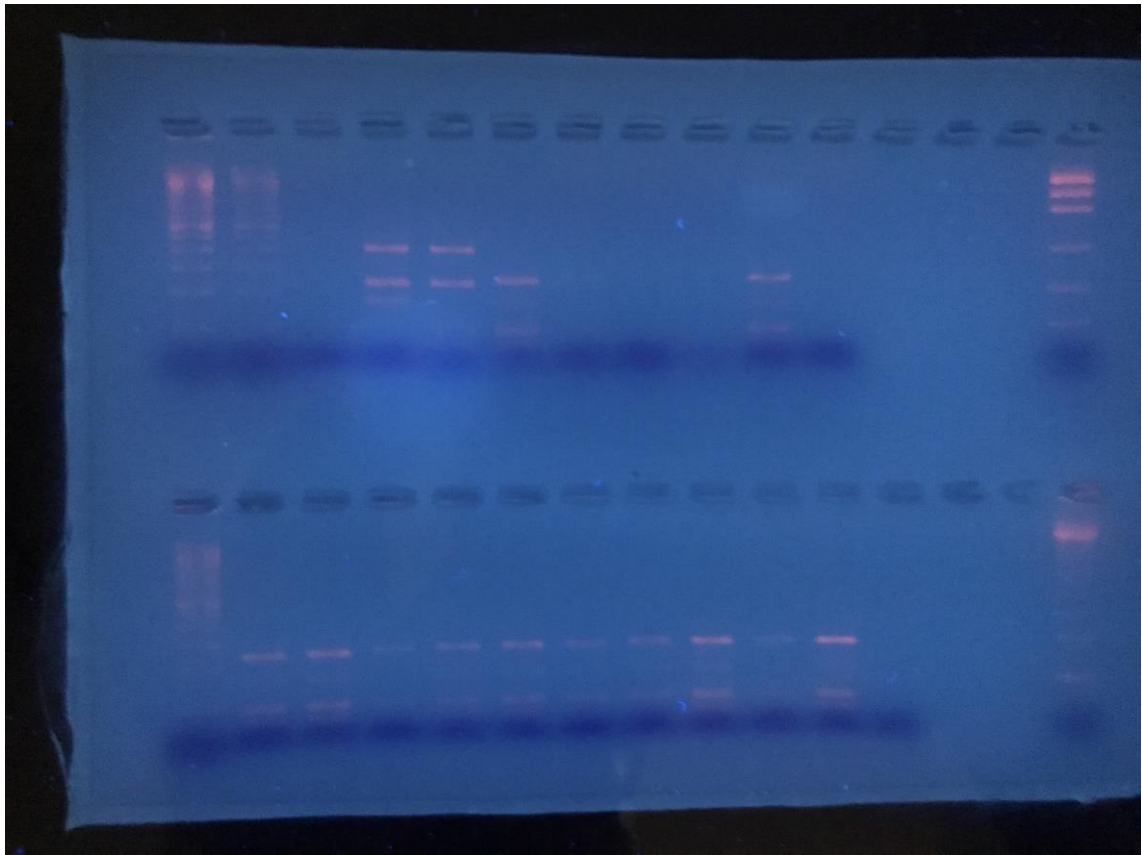

S1 figure.

Image taken with the cell phone camera.

Supplement: S1 Raw images — (PDF) [file pone.0265192.s009.pdf]
