# Supplementary material for: miRNA-21 regulates CD69 and IL-10 expression in canine leishmaniasis
Source: PLoS One. 2022 Mar 24;17(3):e0265192. doi: 10.1371/journal.pone.0265192 (PMC8947396; doi:10.1371/journal.pone.0265192)
Supplement: S1 Table — (DOCX) [file pone.0265192.s005.docx]

**S1 Table.** Biochemical profile of CanL and control groups.

| Animal | Albumin (g/dL) | Total Protein (g/dL) | Globulin (g/dL) | ALT (U/L) | Alkaline phosphatase (U/L) | GGT (mg/dL) | Creatinine (mg/dL) | Urea (mg/dL) |
| --- | --- | --- | --- | --- | --- | --- | --- | --- |
| Reference | 2,6-3,3 | 5,4-7,1 | 2,7-4,4 | 21-102 | 20-156 | 1,2-6,4 | 0,5-1,5 | 10,03-50,03 |
| Infected 1 | 1,6 | 10,4 | 8,8 | 26 | 66 | 5,9 | 0,6 | 42 |
| Infected 2 | 1 | 7,6 | 6,6 | 42 | 243 | 2,9 | 0,7 | 33 |
| Infected 3 | 1,6 | 8 | 6,4 | 26 | 120 | 2 | 0,8 | 32 |
| Infected 4 | 1,8 | 6,1 | 4,3 | 36 | 43 | 1,2 | 0,7 | 28 |
| Infected 5 | 0,94 | 7 | 6,1 | 21 | 29 | 4,9 | 0,8 | 70 |
| Infected 6 | 1,1 | 6,3 | 5,2 | 21 | 109 | 1,9 | 1 | 60 |
| Infected 7 | 1,9 | 8,7 | 6,8 | 92 | 92 | 1 | 0,7 | 49 |
| Infected 8 | 2,2 | 6,8 | 4,6 | 70 | 70 | 1,5 | 0,5 | 35 |
| Infected 9 | 1 | 8,4 | 7,4 | 25 | 135 | 1,9 | 1,5 | 87 |
| Infected 10 | 1,2 | 8,8 | 7,6 | 30 | 42 | 1,4 | 1,5 | 120 |
| Control 1 | 3,3 | 7 | 3,7 | 80 | 74 | 2,6 | 0,9 | 25 |
| Control 2 | 2,9 | 6,7 | 3,8 | 105 | 148 | 5,9 | 0,7 | 30 |
| Control 3 | 3,4 | 6,9 | 3,5 | 40 | 36 | 2,9 | 1 | 27 |
| Control 4 | 2,9 | 7 | 4,1 | 32 | 33 | 1,2 | 1 | 50 |
| Control 5 | 2,7 | 6,6 | 3,9 | 50 | 85 | 1,9 | 1,1 | 21 |

¶ALT: alanine aminotransferase; GGT: gamma glutamyl transferase
