# Supplementary material for: miRNA-21 regulates CD69 and IL-10 expression in canine leishmaniasis
Source: PLoS One. 2022 Mar 24;17(3):e0265192. doi: 10.1371/journal.pone.0265192 (PMC8947396; doi:10.1371/journal.pone.0265192)
Supplement: S2 Table — (DOCX) [file pone.0265192.s006.docx]

**S2 Table**. Complete Blood Count of CanL and control groups.

| Animal | Red blood cells (x10¹²/L) | Hemoglobin (g/dL) | GV (%) | MCV (fL) | MCHC (%) | Leukocytes (x10^9^/L) | Neutrophils (x10^6^/L) | Lymphocytes (x10^6^/L) | Monocytes (x10^6^/L) | Eosinophils (x10^6^/L) | Basophils (x10^6^/L) | Platelets (x1000) | PP (g/dL) |
| --- | --- | --- | --- | --- | --- | --- | --- | --- | --- | --- | --- | --- | --- |
| Reference | 5,5-8,5 | 12,0-18,0 | 37-55 | 60-77 | 32-36 | 6,0-17,0 | 3000-11500 | 1000-4800 | 150-1350 | 150-1250 | raros | 160-440 | 6,0-8,0 |
| Infected 1 | 4,25 | 10,1 | 29 | 68,24 | 34,83 | 5,4 | 4212 | 594 | 486 | 108 | 0 | 220 | 12 |
| Infected 2 | 3,88 | 8,6 | 26 | 67,01 | 33,08 | 13,3 | 11172 | 1995 | 133 | 0 | 0 | 160 | 9 |
| Infected 3 | 3,84 | 9,1 | 25 | 65,1 | 36,4 | 17,9 | 13783 | 2506 | 1253 | 358 | 0 | 400 | 8,4 |
| Infected 4 | 5,38 | 11 | 31 | 57,62 | 35,48 | 16,2 | 11178 | 3402 | 1296 | 324 | 0 | 300 | 7 |
| Infected 5 | 2,95 | 6,5 | 18 | 61,02 | 36,11 | 9,3 | 6417 | 1860 | 837 | 186 | 0 | 280 | 8,2 |
| Infected 6 | 4,3 | 9,5 | 27 | 62,79 | 35,19 | 7,2 | 4320 | 1800 | 288 | 72 | 0 | 140 | 7 |
| Infected 7 | 4,06 | 10 | 28 | 68,97 | 35,71 | 8,5 | 6035 | 2040 | 425 | 0 | 0 | 200 | 10 |
| Infected 8 | 2,1 | 4,1 | 12 | 57,14 | 34,17 | 3 | 2100 | 780 | 30 | 90 | 0 | 220 | 7 |
| Infected 9 | 2,82 | 6,6 | 20 | 70,92 | 33 | 12,4 | 9176 | 2976 | 248 | 0 | 0 | 160 | 8,8 |
| Infected 10 | 3,81 | 8,7 | 25 | 65,62 | 34,8 | 14,6 | 10950 | 3358 | 146 | 146 | 0 | 280 | 9 |
| Control 1 | 7,89 | 17,8 | 53 | 67,17 | 33,58 | 16,2 | 10692 | 2754 | 1496 | 1250 | 0 | 200 | 8 |
| Control 2 | 6,78 | 16,9 | 49 | 72,27 | 34,49 | 15,7 | 10048 | 2983 | 628 | 2041 | 0 | 300 | 7,8 |
| Control 3 | 7,89 | 17,8 | 52 | 65,91 | 34,23 | 12,2 | 6588 | 4800 | 610 | 122 | 0 | 320 | 7,8 |
| Control 4 | 6,78 | 17,3 | 50 | 73,75 | 34,6 | 16,2 | 10854 | 3726 | 324 | 1296 | 0 | 220 | 8 |
| Control 5 | 7,44 | 17,1 | 51 | 68,55 | 33,53 | 10,3 | 6077 | 3605 | 515 | 103 | 0 | 220 | 6,6 |

¶GV: globular volume; MCV: mean corpuscular volume; MCHC: mean corpuscular hemoglobin concentration; PP: plasma protein
