# Supplementary material for: miRNA-21 regulates CD69 and IL-10 expression in canine leishmaniasis
Source: PLoS One. 2022 Mar 24;17(3):e0265192. doi: 10.1371/journal.pone.0265192 (PMC8947396; doi:10.1371/journal.pone.0265192)
Supplement: S3 Table — (DOCX) [file pone.0265192.s007.docx]

**S3 Table.** **CD69 expression in lymphocytes.** Mean±SD of CD69 in CD4+, CD8+ and CD21+ cells measured by flow cytometry in splenic leukocytes of CanL group after transfection with miR-21 mimics and inhibitors for 48h at 37ºC and 5% CO_2_.

| Lymphocytes | Mimic (mean± SD) | Inhibitor (mean±SD) | Scrambled (mean±SD) | P value* |
| --- | --- | --- | --- | --- |
| CD69+CD4+ | 22955±8768 | 19051±9307 | 23076±8611 | 0.5216 |
| CD69 +CD8+ | 36462±26928 | 21607±8231 | 31327±12309 | 0.0934 |
| CD69+CD21+ | 41801±26658 | 52115±35065 | 69723±37559 | 0.0394 |

*p value considering Friedman test
