# Supplementary material for: miRNA-21 regulates CD69 and IL-10 expression in canine leishmaniasis
Source: PLoS One. 2022 Mar 24;17(3):e0265192. doi: 10.1371/journal.pone.0265192 (PMC8947396; doi:10.1371/journal.pone.0265192)
Supplement: Attachment — Submitted filename: Response to Reviewers.docx [file pone.0265192.s010.docx]

Response to Reviewers

Manuscript No.: PONE-D-21-21939

**Title:** miRNA-21 regulates CD69 and IL-10 expression in canine leishmaniasis

Dear Editor,

We appreciate the careful review of our manuscript by the reviewers. Their constructive comments and suggestions are much appreciated, and the new version of the manuscript has benefited considerably from their assistance Please note that all changes made in the text are highlighted in red.

Editor Comment:

**The authors should provide experiments and controls for the transfection efficacy of the micro RNA to be sure that the effect was due to the action or not of the transfected micro RNA;**

R: The experiment that represents transfection efficacy was added to supporting information;.

Reviewer Comments:

Author’s answer to Reviewer Comments: We thank the referee for providing constructive comments to improve the article. All criticisms and detailed corrections have been addressed and the following revisions have been made.

We have accepted all the suggestions and requests made by the Editor and Reviewers in the manuscript.

Sincerely yours,

Valéria Marçal Felix de Lima, MSc, PhD

São Paulo State University – UNESP

Reviewer #1

Author’s answer: We thank the referee for providing constructive comments to improve the article. All criticisms and detailed corrections have been addressed and the following revisions have been made.

Reviewer #1’s comments	Answers

**Table S1, please add this table as main table 1**

R: Table 1 was put as main table as recommended.

**Figure 1: Please indicate in the Y-axis “fold change of miRNA 21”. How the fold change of miRNA 21 expression was calculated is not completely clear. Would you please clarify this information in the figure legend?**

R: The required information was added to the figure legend.

**Figure 2: Please indicate reference description in Figure 2 for blue and red histograms. In the result section, please correct the title of the figure, as “****Expression of miR-21 targets gene in splenic leukocytes of CanL and healthy dogs”. Same comment for figure legend 2**

R: The information was modified in the text.

**Figure 3: Please indicate in Figure legend 3 title, transfection of what is referring to. In the result section, indicate the reason for using miRNA mimics and inhibitors. Have you been waiting for opposite results?**

R: The legend of the figure was modified. We used both mimics and inhibitor because increased and decreased expression could facilitate the understanding of how miR-21 does the regulation.

**Figure 4: Please also include data of CD4 and CD8 in Figure 4.**

R: The figure was added.

**Discussion section. Even though your results showed no changes in the regulation of FAS, FASL, and CCR7 by miRNA 21, these results could be related to low transfection efficacy, not adequate concentration range of mimic or inhibitor, among others. Did you try different concentrations? Please discuss these aspects very carefully and include them as limitations of the work**.

R: We tested all the different concentrations recommended by the manufacturer in order to optimize the transfection rate, and used the concentrations that gave the best results.

**This sentence is unclear; please revise “FAS and FASL play critical roles in the immune system, particularly in the death of target cells infected by pathogens and autoreactive lymphocytes' death.**

R: This sentence was reformulated.

**Same comment as above regarding the effect of miRNA 21 mimic and inhibitor on CD4 and CD8. Did you perform a doses resp**

R: Yes, we tested different doses to optimize the results.

Reviewer #2

Author’s answer: We thank the referee for providing constructive comments to improve the article. All criticisms and detailed corrections have been addressed and the following revisions have been made.

Reviewer #2’s comments	Answers

**Line 101: The authors stated that “Splenic fragments in control dogs were removed by surgical excision [25]”. I wish to understand if the Animal Ethics Committee approved the collection of splenic fragments from healthy animals. If yes, could you please clarify in which circumstances? Please, provide the Animal Ethics certificate. Also, it is unclear why reference 25 was used.**

R: Animal Ethics Committee approved the collection of splenic fragments from healthy animals. The owners assigned a term of consentient authorizing the procedure. Reference 25 was used to describe the surgical procedure.

**Line 105: Please, explain how the fragments were macerated.**

R: The information was added to the text.

**Line 110: Please provide the description or the commercial reference for the red blood cell lysis buffer**

R: The information was added to the text.

**Line 136: Confirm if the DNA was extracted from cells or directly from tissue**

R: DNA was extracted from cells.

**Line 157: The authors stated “To confirm that miR-21 is upregulated in dogs with CanL obtained by [11],” Please, explain why reference 11 was used.**

R: Reference 11 was used to show that the waiting result has already been observed in other paper.

**Line 169: A pool of which cDNAs was used for normalization - control, infected, or both? Please explain where the absolute quantification of miR-21 was used.**

R: A pool of both cDNAs. Absolute quantification was used in all samples of both groups.

**Line 173: Please, insert the reference or complete description for SNORD96A.**

R: The information was added to the text.

**Results**

**Figure 1 legend: Please, insert the number of animals (samples) analyzed.**

**Figure 2 legend: Please, detail which range is referring, min-max, SD? I suggest presenting the control group first in the graph (same order as in figure 1).**

**Figures 4 and 5 legends: Please, detail which range is referring, min-max, SD?**

R: All modifications were done.

**I am concerned about the validation of the transfection. The authors did not show a control condition for the experiment (a condition with the transfection reagent only, without the scrambled sequence). Did you perform a pilot experiment with i.e., cells treated with transfection reagent only? Also, it is mandatory to validate the expression of miR-21 after transfection. How do the authors know the transfection efficiency relative to miR-21 inhibition? Is it possible to run an experiment to evaluate the miRNA expression after transfection? Do the authors have frozen samples? Gene expression should be similar in both untransfected cells and cells transfected with the negative control. Comment, please.**

According the manufacturer, “The expression of an endogenous gene, which is known to be a target of the miRNA under study, is measured after mimic/inhibitor transfection. The effect of the mimic/inhibitor is determined by comparing this result with the gene expression in untransfected cells or cells transfected with a negative control. Gene expression is often measured at the protein level, for example, by western blot, as miRNAs often inhibit translation of their target genes and do not cause degradation of the target transcript. This means that the effect of a miRNA mimic or inhibitor can often not be determined using quantitative, real-time PCR.”

**Besides that, the average CD69 expression in the control group was around 40000 (figure 2). When the cells of this group were transfected with the scrambled sequence, the average expression was around 75000 (figure 3). It seems that the scrambled condition is increasing the expression. At the same time, the inhibitor condition is achieving an expression around 40000, similar to the control group expression in figure 2. Results from the negative control should be compared to results from untransfected cells. It seems that adequate controls are missing. Comment, please.**

Although the numbers are different, the analysis was performed and there was no statistical difference.

According to manufacturer, “A negative control should be transfected in every experiment and will indicate if results are nonspecific. Comparison of results from the negative control with results from the miRNA mimic under study can be used to confirm that the observed results are specific to the miRNA mimic under study.”

The graph below represents the control group containing comparative analyzes with non-transfected cells and using only the transfection reagent (HiperFect), demonstrating that there was no statistical difference between the other groups.

**The authors found significantly less expression of CD69 in control and canL groups when miR-21 was mimic on the other side the inhibition did not produce the opposite effect. Comment, please.**

The amount of miR-21 present in the samples can be so high that the concentration used in the inhibitor and indicated by the manufacturer was not enough to show an opposite effect.

**Discussion:**

**Line 334-335: Maybe the low transfection rate of this study does not support the previous sentence “suggesting that, in dogs, these proteins may not be targets of miR-21.” Please, comment.**

The low transfection rate obtained may not have been sufficient to modulate the expression of these molecules.

In Other experimental models, these proteins are targets of miR-21:

1- Liu Y, Ren L, Liu W, Xiao Z. MiR-21 regulates the apoptosis of keloid fibroblasts by caspase-8 and the mitochondria-mediated apoptotic signaling pathway via targeting FasL. Biochem Cell Biol. 2018 Oct;96(5):548-555. doi: 10.1139/bcb-2017-0306. Epub 2018 Mar 10. PMID: 29527928.

2- Shang C, Guo Y, Hong Y, Liu YH, Xue YX. MiR-21 up-regulation mediates glioblastoma cancer stem cells apoptosis and proliferation by targeting FASLG. Mol Biol Rep. 2015 Mar;42(3):721-7. doi: 10.1007/s11033-014-3820-3. Epub 2014 Nov 14. PMID: 25394756.

3- Marega LF, Teocchi MA, Dos Santos Vilela MM. Differential regulation of miR-146a/FAS and miR-21/FASLG axes in autoimmune lymphoproliferative syndrome due to FAS mutation (ALPS-FAS). Clin Exp Immunol. 2016 Aug;185(2):148-53. doi: 10.1111/cei.12800. Epub 2016 May 24. PMID: 27060458; PMCID: PMC4954998.

4- Sayed D, He M, Hong C, Gao S, Rane S, Yang Z, Abdellatif M. MicroRNA-21 is a downstream effector of AKT that mediates its antiapoptotic effects via suppression of Fas ligand. J Biol Chem. 2010 Jun 25;285(26):20281-90. doi: 10.1074/jbc.M110.109207. Epub 2010 Apr 19. PMID: 20404348; PMCID: PMC2888441.
