# Supplementary material for: miRNA-21 regulates CD69 and IL-10 expression in canine leishmaniasis
Source: PLoS One. 2022 Mar 24;17(3):e0265192. doi: 10.1371/journal.pone.0265192 (PMC8947396; doi:10.1371/journal.pone.0265192)
Supplement: Attachment — Submitted filename: 20211022_Plos_revision.docx [file pone.0265192.s011.docx]

The authors answered all points addressed by the reviewer. Unfortunately, the authors were not able to solve the main issue raised by the reviewer. Although the author affirms that there is no difference among scrambled, untransfected, and HiperFect conditions, only in two samples in the scrambled condition the CD69 expression is high. So, my point is that the mimic is not decreasing the expression, but something happens in the scrambled that resulted in a higher expression. I still wish to see the raw data of all of them.
