# Supplementary material for: miRNA-21 regulates CD69 and IL-10 expression in canine leishmaniasis
Source: PLoS One. 2022 Mar 24;17(3):e0265192. doi: 10.1371/journal.pone.0265192 (PMC8947396; doi:10.1371/journal.pone.0265192)
Supplement: Attachment — Submitted filename: Response to Reviewers.docx [file pone.0265192.s012.docx]

Response to Reviewers

Manuscript No.: PONE-D-21-21939

**Title:** miRNA-21 regulates CD69 and IL-10 expression in canine leishmaniasis

Dear Editor,

We appreciate the careful review of our manuscript by the reviewers. Their constructive comments and suggestions are much appreciated, and the new version of the manuscript has benefited considerably from their assistance Please note that all changes made in the text are highlighted in red.

Reviewer Comments:

Author’s answer to Reviewer Comments: We thank the referee for providing constructive comments to improve the article. All criticisms and detailed corrections have been addressed and the following revisions have been made.

We have accepted all the suggestions and requests made by the Reviewers in the manuscript.

Sincerely yours,

Valéria Marçal Felix de Lima, MSc, PhD

São Paulo State University – UNESP

Reviewer #1

Author’s answer: We thank the referee for providing constructive comments to improve the article. All criticisms and detailed corrections have been addressed and the following revisions have been made.

Reviewer #1’s comments	Answers

**Figure 1: Include “fold change of miRNA 21” on the Y-axis of the Graph. This will help the reader to quickly understand what fold changes are referring to in that graph.**

R: The y-axis was renamed as recommended.

**Figure 4: The Y-axis of the 3 Graphs showing data of CD69 expression on CD4, CD8, and CD21 positive cells are incorrectly labeled. Those graphs should state “Mean Fluorescence Intensity of CD69 on CD4+ cells, or CD8+ cells or CD21+ cells” instead of the current labels that are very confusing, Mean CD8+ cells (FL1)/CD69+ cells (FL2). Please correct accordingly.**

R: The Y-axis title was modified.

**S2 Fig. It is not clear what the authors are measuring as the control of transfection. What do more dead cells (7ADD+) mean in regard to transfection efficacy? please clarify this point in result section.**

R: The information to better explain the transfection rates was added in the methods section.

Reviewer #2

Author’s answer: We thank the referee for providing constructive comments to improve the article. All criticisms and detailed corrections have been addressed and the following revisions have been made.

Reviewer #2’s comments	Answers

**The authors answered all points addressed by the reviewer. Unfortunately, the authors were not able to solve the main issue raised by the reviewer. Although the author affirms that there is no difference among scrambled, untransfected, and HiperFect conditions, only in two samples in the scrambled condition the CD69 expression is high. So, my point is that the mimic is not decreasing the expression, but something happens in the scrambled that resulted in a higher expression. I still wish to see the raw data of all of them**. **I strongly suggest a statistical review as in my checking (with the raw data available) there were no defferences in CD69 expression in control and CanL groups (ANOVA followed by Dunn's multiple comparison test).**

CD69 expression in untransfected cells in CanL and Control group

Normality test (don’t pass)

Non parametric test to compare the expression in CanL and Control group (Mann Whitney test).

Analysis post transfection with mimics and inhibitors of miR-21:

CanL group data

Normality test (don’t pass)

Friedman test (non parametric)

Post test (Dunn’s multiple comparisons)

Control Group data

Normality test

Friedman test

Dunn’s multiple comparison test
