# Supplementary material for: miRNA-21 regulates CD69 and IL-10 expression in canine leishmaniasis
Source: PLoS One. 2022 Mar 24;17(3):e0265192. doi: 10.1371/journal.pone.0265192 (PMC8947396; doi:10.1371/journal.pone.0265192)
Supplement: Attachment — Submitted filename: Response to Reviewers.docx [file pone.0265192.s014.docx]

Response to Reviewers

Manuscript No.: PONE-D-21-21939

**Title:** miRNA-21 regulates CD69 and IL-10 expression in canine leishmaniasis

Dear Editor,

We appreciate the careful review of our manuscript by the reviewers. Their constructive comments and suggestions are much appreciated.

The Reviewer 2 asked for a parametric statistical analysis to be performed on the animals in the control group (n=5), but the data did not show normality. Therefore, we maintained our statistic using a non-parametric test.

Reviewer Comments:

Author’s answer to Reviewer Comments: We thank the referee for providing constructive comments to improve the article. All criticisms and detailed corrections have been addressed and the following revisions have been made.

Sincerely yours,

Valéria Marçal Felix de Lima, MSc, PhD

São Paulo State University – UNESP

Reviewer #2

Author’s answer: We thank the referee for providing constructive comments to improve the article. All criticisms and detailed corrections have been addressed and the following revisions have been made.

Reviewer #2’s comments	Answers

**According to the Prism file provided by the authors, the statistical analysis of CD69 expression after transfection must be performed with a parametric test (ANOVA) for the CONTROL group instead of the nonparametric test used. Also, data of HiperFect buffer and untransfected cells must be provided in figure 3. Update methods and results section accordingly**.

R: Figure 3 was modified and HiperFect and untransfected cells data were added.

To claim that our data has a normal distribution, we rigorously pass three different normality tests. Since of the three, only 1 test showed normality in the data, while the other 2 showed that n is too small to present a normal distribution. Given this, we chose to use a non-parametric test, which best represents our data.

Similar experiment has already been published by our research group, using this same statistical test, in the work of MELO, et al., 2019.

Attached are some links to works with values of n similar to ours, which used non-parametric tests.

<https://www.ncbi.nlm.nih.gov/pmc/articles/PMC4029133/>

<https://www.ncbi.nlm.nih.gov/pmc/articles/PMC7023135/>

<https://www.ncbi.nlm.nih.gov/pmc/articles/PMC6905561/>

<https://www.ncbi.nlm.nih.gov/pmc/articles/PMC5962361/>

<https://www.ncbi.nlm.nih.gov/pmc/articles/PMC6962187/>
